# Testing the contagious nature of allopreening: bystander ravens are affected by conspecifics’ affiliative interactions

**DOI:** 10.1016/j.anbehav.2021.12.009

**Published:** 2022-02

**Authors:** Katharina Wenig, Lisa Pacher, Thomas Bugnyar

**Affiliations:** a Department of Behavioural and Cognitive Biology, University of Vienna, Austria; b Haidlhof Research Station, University of Vienna and University of Veterinary Medicine Vienna, Bad Vöslau, Austria

**Keywords:** affiliation, allopreening, behavioural contagion, cognition, emotional contagion

## Abstract

Emotional contagion refers to the mechanism of aligning with conspecifics’ emotional states and is thought to be highly beneficial in social group living. While emotional contagion is well studied in humans, most studies in nonhuman animals fail to clearly distinguish between behavioural and emotional contagion. Furthermore, evidence for positive emotional contagion in nonhuman animals is almost entirely restricted to the context of play. In the present study, we aimed at adding observational evidence of contagion in a positive context, while separating aspects of behavioural and emotional contagion. In a group of nonbreeder common ravens, *Corvus corax*, we investigated whether witnessing conspecifics in positive social interaction, namely allopreening, would influence a bystander’s behavioural and, possibly, emotional state. We recorded behavioural expressions of bystanders in postpreening observation phases and compared them to those in matched-control observation phases. We found effects of witnessing others’ allopreening on the bystanders’ subsequent affiliative interactions but not on their self-directed behaviours (e.g. autopreening) or agonistic interactions. Specifically, bystanders were more likely to engage in allopreening themselves in the postpreening observation phase than in the matched-control observation phase, which could be explained via behavioural and emotional contagion; however, bystanders also showed elevated levels of nonpreening affiliative interactions and spent more time close to conspecifics after observing others allopreening, which hints towards a more general effect on the bystanders’ emotional states. Whether these nonpreening affiliative interactions are indeed an indication of emotional contagion needs to be tested in further studies that measure, and manipulate, emotional states.

Perceiving, evaluating and reacting to conspecifics’ emotional states are important challenges of social group living (Ferretti & Papaleo, 2019; Pérez-Manrique & Gomila, 2021) and particularly crucial for the transfer of information (Briefer, 2018; Decety et al., 2016; Plutchik, 1987; Preston & De Waal, 2002), coordination and cohesion between group members (Briefer, 2018; De Vignemont & Singer, 2006; Decety et al., 2012, 2016; Pérez-Manrique & Gomila, 2018). One way to achieve an emotional alignment with another individual is via emotional contagion (Hatfield et al., 1993). However, few studies have addressed emotional contagion in nonhuman species (see Adriaense et al., 2020 and Pérez-Manrique & Gomila, 2021 for reviews) and these have typically made use of negatively valenced stimuli to see whether and how emotional states would transfer between individuals. Positive emotional contagion, on the other hand, is a phenomenon most studied and most evident in humans: interacting with cheerful individuals or witnessing friendly third-party interactions leads to positive emotional states in bystanders as well as friendly behaviour towards others (Nook et al., 2016; Pugh, 2001; Schnall et al., 2009). In recent behavioural experiments in nonhuman animals (henceforth ‘animals’), demonstrator pigs, *Sus scrofa*, have been shown to express a positive emotional state after being granted access to an enriched environment and to consequently transfer their positive emotional state to conspecifics (Reimert et al., 2017). However, the best indications of positive contagion come from studies on animal play. Playbacks of play vocalization in New Zealand parrots, *Nestor notabilis*, resulted in elevated play rates in conspecifics (Schwing et al., 2017). Osvath and Sima (2014) and Wenig et al. (2021) showed that observing others playfully manipulating objects transferred to locomotion and social play in young common ravens, *Corvus corax*. Furthermore, studies on captive rats, *Rattus norvegicus*, revealed that social play increased when a playful individual was introduced to a less playful one (Pellis & McKenna, 1992; Varlinskaya et al., 1999). Rapid facial mimicry, an involuntary and automatic response, was recorded in a play context in gelada baboons, *Theropithecus gelada*, and orang-utans, *Pongo pygmaeus* (Davila Ross et al., 2008; Mancini et al., 2013). However, these latter studies on animal play, strictly speaking, showed behavioural contagion and concluded an effect on the bystanders’ emotional state without testing for it.

Allogrooming offers another suitable context to study positive emotional contagion as it plays a role not only in ectoparasite removal on inaccessible body parts (Barton, 1985; Freeland, 1976) but as an affiliative behaviour also in social bonding and social communication (Dunbar, 1991; Emery et al., 2007; O’Brien, 1993; Radford & Du Plessis, 2006; Seyfarth & Cheney, 1984). Receiving grooming and grooming others have been shown to lead to a reduction in heart rate, glucocorticoid release and self-directed behaviours in a variety of animals such as primates (e.g. in chimpanzees, *Pan troglodytes*, rhesus macaques, *Macaca mulatta*, long-tailed macaques, *Macaca fascicularis*, crested macaques, *Macaca nigra*, Barbary macaques, *Macaca sylvanus*, and pigtailed macaques, *Macaca nemestrina*; Aureli et al., 1999; Aureli & Yates, 2010; Boccia et al., 1989; Crockford et al., 2013; Keverne et al., 1989; Schino et al., 1988; Shutt et al., 2007), horses, *Equus caballus* (Feh & de Maziè res, 1993) and birds (e.g. green woodhoopoes, *Phoeniculus purpureus*: Radford & Du Plessis, 2006; common ravens: Stöwe et al., 2008). Allogrooming in Barbary and rhesus macaques has recently been assessed as a potential study context for positive contagion (Berthier & Semple, 2018; Ostner et al., 2021). Observers of allogrooming interactions in groups of females were subsequently more likely to initiate and engage in allogrooming themselves, compared to control observations. Ostner et al. (2021) further assessed social factors of visual grooming contagion in rhesus macaques, showing that high-ranking observer individuals engaged in allogrooming more rapidly than lower-ranking individuals, while relationship quality between demonstrators and observers did not affect the latency to allogroom. Barbary macaques (Berthier & Semple, 2018) not only engaged in more grooming and nongrooming affiliative interactions after witnessing others allogrooming, but also showed lower rates of self-directed behaviours. In a study on common marmosets, *Callithrix jacchus*, subjects were exposed to videos of allogrooming conspecifics and consequently also showed increased levels of allogrooming but no signs of decreased self-directed behaviours (Watson, 2011).

Allopreening, the avian equivalent to allogrooming, is a typical behaviour observed in pair-bonded and/or group-living bird species (Harrison, 1965; Morales Picard et al., 2020; Sparks, 1964). Common ravens belong to this category; they are renowned for engaging in a range of affiliative interactions, including allopreening (Gwinner, 1964). In the present study, we tested whether others’ allopreening interactions were contagious in a captive group of ravens. Following Berthier and Semple (2018), we also used an adaption of the well-established postconflict/matched-control observation method (de Waal & Yoshihara, 1983) to assess the ravens’ behaviour after witnessing conspecifics’ allopreening interactions (postpreening, PP observation) in comparison to matched-control (MC) observations. Specifically, we tested the hypothesis that observing conspecifics in allopreening interactions would influence the bystanders’ subsequent behavioural expressions and their emotional states. We expected bystanders to show behavioural matching, indicated by an increase in preening, which could be either self-directed (i.e. autopreening) or other-directed (i.e. allopreening). We also expected an increase in allopreening-related activities, such as approaching conspecifics, spending time close to conspecifics, engaging in nonpreening affiliative interactions, e.g. touching a conspecific with the beak, which could be interpreted as an indication of emotional contagion (compare Osvath & Sima, 2014 for a similar argument in play) or as incomplete expressions of allopreening attempts (compare Ostner et al., 2021). Finding fewer agonistic interactions and (nonpreening) self-directed behaviours, i.e. behavioural expressions that are of different valence and comprising rather distinct sets of motor patterns compared to preening, would be a further indication of emotional contagion. We relied exclusively on naturally occurring behaviours, assuming that allopreening is associated with a positive emotional state and agonistic behaviour expressions as well as nonpreening expressions of self-directed behaviours are associated with a negative emotional state (compare Munteanu et al., 2017).

## Methods

### Animals and Housing

**Figure 1:**
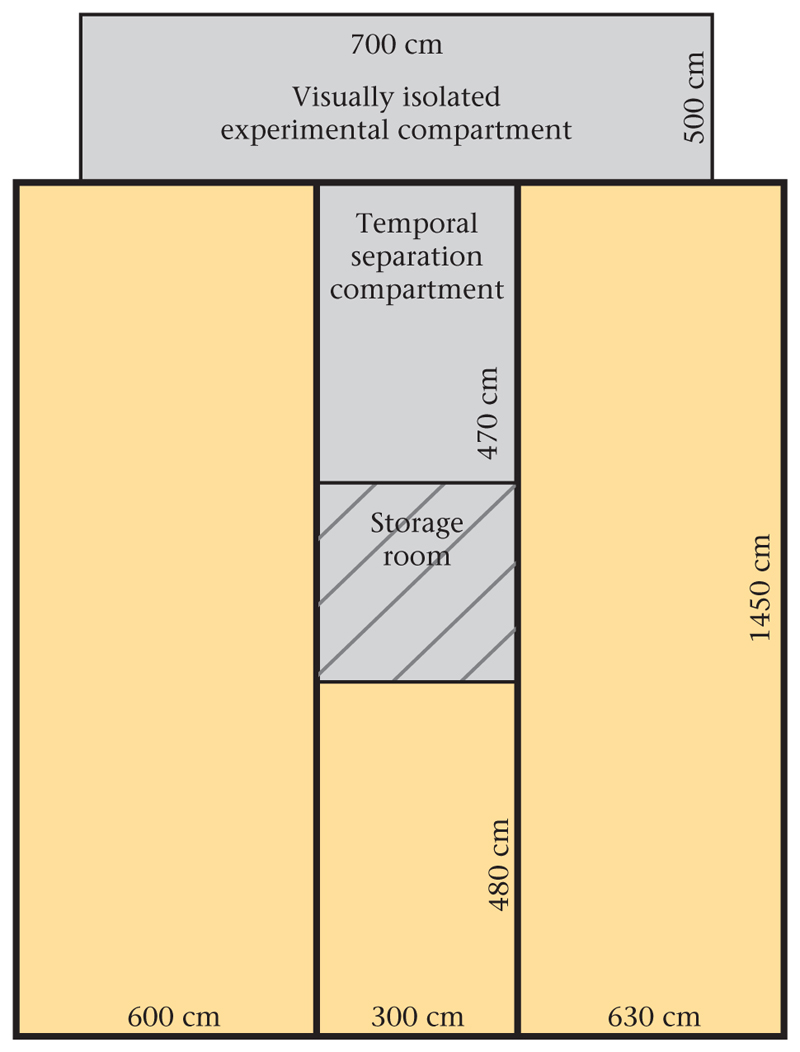
Sketch of the nonbreeder ravens’ home aviary, depicting compartment sizes. Yellow shaded areas indicate compartments to which ravens had access during data collection (193 m^2^). White areas (experimental and separation compartments) were additionally accessible as part of the home aviary outside of data collection.

We observed a captive group of nonbreeder common ravens at the Haidlhof research station in Bad Vöslau, Lower Austria. The housing schedule in the 242 m^2^ outdoor aviary (Fig. 1) was designed to simulate natural social groupings of nonbreeder ravens (Braun & Bugnyar, 2012; Marzluff et al., 1996). During the period of data collection, the captive nonbreeder group was composed of 10–16 individuals of different age classes, with nine subadult group members (seven females, two males) forming the stable core of the group. Focal data were collected on these nine core group members while all group members (dynamically ranging between 10 and 16 individuals) could serve as allopreening demonstrators. During data collection, the visually restricted areas of the aviary were closed off. Hence, all nonbreeder ravens had access to a defined area (193 m^2^, Fig. 1), which ensured the human observer could see all birds. When perched, ravens could see each other from several parts of the defined area in the aviary.

All nine focal subjects hatched in April 2018 and originated from five breeding pairs of our captive colony. At the age of 3–4 weeks, they were taken out of their parents’ nests and hand raised by humans under standardized conditions. At 6 weeks, shortly before the chicks fledged, nests were transferred to the outdoor aviary. After fledging, subjects were introduced stepwise to older ravens (four females, three males) and thus became members of the nonbreeder group. Upon pair formation, adult ravens were taken out of the nonbreeder group and housed in pairs either at Haidlhof research station or at other institutions, resulting in a dynamic change of the group’s size and composition.

Since the majority of ravens were hand raised by humans, they were naturally used to their presence inside and outside the aviary. The remaining parent-raised individuals were also familiar with being observed from outside the aviary due to the daily research routines. All individuals were marked with a coloured leg band for identification.

All aviaries were equipped with natural structures (e.g. wood, rocks, gravel, sand) and artificial objects (e.g. food bowls, bathing pools, toys) to promote a variety of behaviour expressions (e.g. exploration, manipulation and caching of food and objects, conflict escape possibilities) and to provide protection during extreme weather conditions. All ravens were kept on a diet of meat, eggs, vegetables, dairy products, bread and phytobiotics. Water was provided ad libitum.

### Data Collection

Behavioural observations were conducted between 0900 and 1500 by four human observers from outside the aviary. Before the data collection started, ravens were habituated to the presence of the human observers for 2 days. The procedure used for data collection was adapted from the well-established postconflict/matched-control method, first used by de Waal and Yoshihara (1983), as shown in Berthier and Semple (2018).

An observation session began when one individual (henceforth ‘focal subject’) witnessed (i.e. oriented visually towards) an allopreening interaction between two conspecifics for at least 5 s and from a maximum distance of 5 m. The postpreening (PP) observation on the focal subject started once the allopreening interaction was terminated, or the focal subject moved away from the allopreening interaction (> 5 m). Within the PP observation, we recorded all self-directed behaviours (beak wipe, scratch, stretch, shake, autopreening; see Appendix Table A1) as well as social behaviours, namely agonistic (chase, displace, threat, fight; see Table A1) and affiliative behaviours (contact-sit, touch/hold, allopreening; see Table A1) with involvement of the focal individual. In addition, we recorded the number of approaches of the focal subject towards a conspecific as well as how long the focal subject spent close to at least one conspecific (< 1 m). The PP observation lasted until the focal subject became involved in an allopreening bout itself or for a maximum of 10 min, if no allopreening interaction occurred. For every protocol, we recorded date, time and group composition of the PP observation.

On the next (or next possible) day, a matched-control (MC) observation on the same focal subject was recorded at the same time (within a 1 h range), given the same group composition and for the same length as the corresponding PP observation. This way we controlled not only for the time of day and the birds’ respective activity levels but also for the presence of bystanders. Prior to the MC observation, the focal subject was observed for 10 min to ensure no involvement in affiliative or agonistic interactions occurred. If an allopreening interaction involved the focal subject during the 10 min before the observation phase, another PP observation was conducted, and the MC observation was delayed to the following day. In the MC observation, we recorded the same behaviours as during the PP observation.

All PP and MC observations were videorecorded from outside the aviary, with comments on the individuals involved for easier identification during video analysis. Overall, we aimed at collecting a minimum of three PP/MC pairs per focal subject within the given time frame of the data collection period. Prior power analysis was not conducted.

### Video Analysis

MC observations were recorded for the same length as the corresponding PP observation or a maximum of 10 min. However, if allopreening in the MC observation occurred earlier than in the PP observation, the observation time of the PP was shortened, aligning both observations to the same lengths. The resulting video material was coded in line with an established ethogram (see Appendix Table A1), using Loopy (Loopbio, Vienna, Austria). A second coder reviewed 10% of the video material, with agreement rates for detection and subsequent classification of behaviours between both coders of above 90%.

### Data Analysis

Following de Waal and Yoshihara (1983), PP/MC pairs were classified in three categories: if an allopreening bout occurred in the PP observation but not in the corresponding MC, the PP/MC pair was classified as ‘attracted’; if an allopreening bout occurred in the MC observation but not in the corresponding PP, the PP/MC pair was classified as ‘dispersed’; if no allopreening occurred in either of the two corresponding observations, the PP/MC pair was labelled as ‘neutral’. Different behavioural expressions were aggregated into broader categories (e.g. chase, displace, threat, fight into category ‘agonistic behaviours’, see Appendix Table A1) while preening-related behaviours were excluded from their respective categories (allopreening from affiliative behaviours, autopreening from self-directed behaviours) and analysed separately.

To avoid pseudoreplication, individual averages per focal subject were calculated and consequently used to test for differences between attracted versus dispersed PP/MC pairs as well as for differences between PP and MC with regard to the time spent close to conspecifics, the time spent in affiliative interaction, the number of approaches towards conspecifics and the number of self-directed and agonistic behaviours. As all variables were not normally distributed according to the Shapiro–Wilk normality test, we calculated nonparametric two-tailed Wilcoxon signed-rank tests on paired samples. Statistical analysis and tests were carried out in R Studio Desktop 1.4.1106 (RStudio Team, 2020).

### Ethical Note

No individuals were ever deprived of food or water. The described housing and testing conditions comply with the ASAB/ABS Guidelines for the Use of Animals in Research, the Austrian government guidelines, and the institutional guidelines of the University of Vienna. The study was approved by the Animal Ethics and Experimentation Board of the Faculty of Life Science, University of Vienna (2020-003). As this study was strictly noninvasive and based purely on observations, it is not classified as an animal experiment under the Austrian Animal Experiments Act.

## Results

### Allopreening

**Figure 2:**
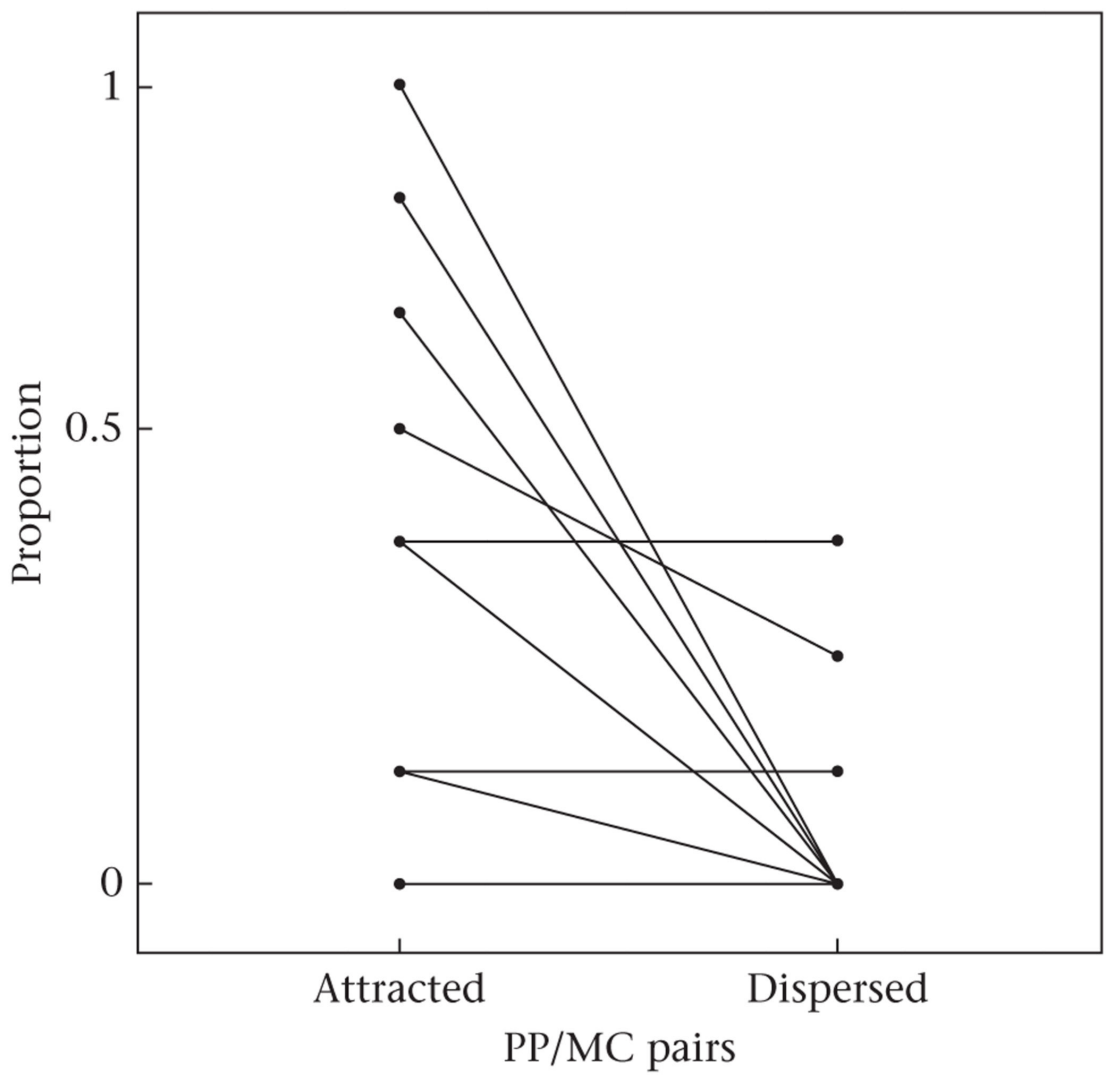
Proportions of ‘attracted’ and ‘dispersed’ PP (postpreening)/MC (matched-control) pairs per focal subject. Attracted: allopreening in the PP but not in the corresponding MC observation. Dispersed: allopreening in the MC but not in the corresponding PP observation. Neutral: no allopreening in PP or in MC observation; neutral PP/MC pairs are not depicted directly but proportions can be calculated by 1–(proportion_attracted_ + proportion_dispersed_). Solid lines indicate female focal subjects; dashed lines indicate male focal subjects. Wilcoxon test: *P* = 0.036, *N* = 9.

In total, 40 PP/MC pairs were collected, comprising data of nine focal individuals (range three to six pairs per individual). Of these 40 PP/MC pairs, 18 were labelled ‘attracted’ (45% of the data set), i.e. allopreening occurred within the PP observation but not in the MC observation; three pairs were labelled ‘dispersed’, as allopreening interactions occurred within the MC observation but not in the PP observation (7.5% of the data set) and 19 PP/MC pairs (47.5% of the dataset) did not contain any allopreening interactions and were therefore categorized as ‘neutral’. A Wilcoxon signed-rank test revealed a significant difference between the proportion of attracted versus dispersed PP/MC pairs (average proportion calculated per focal subject, two-tailed Wilcoxon signed-rank test on paired samples: *V* = 21, *P* < 0.05, effect size *r* = 0.733, *N* = 9), with attracted pairs occurring more frequently than dispersed pairs (median: attracted: 0.33; dispersed: 0), and therefore allopreening being expressed significantly more often within PP than in MC observations (Fig. 2).

In PC observations of attracted pairs, focal subjects engaged in allopreening with a previous demonstrator in five of 18 cases (ca. 27.8%) while they engaged in allopreening with another, previously not involved individual in 13 of 18 cases (ca. 72.2%).

### Social Behaviours

**Figure 3:**
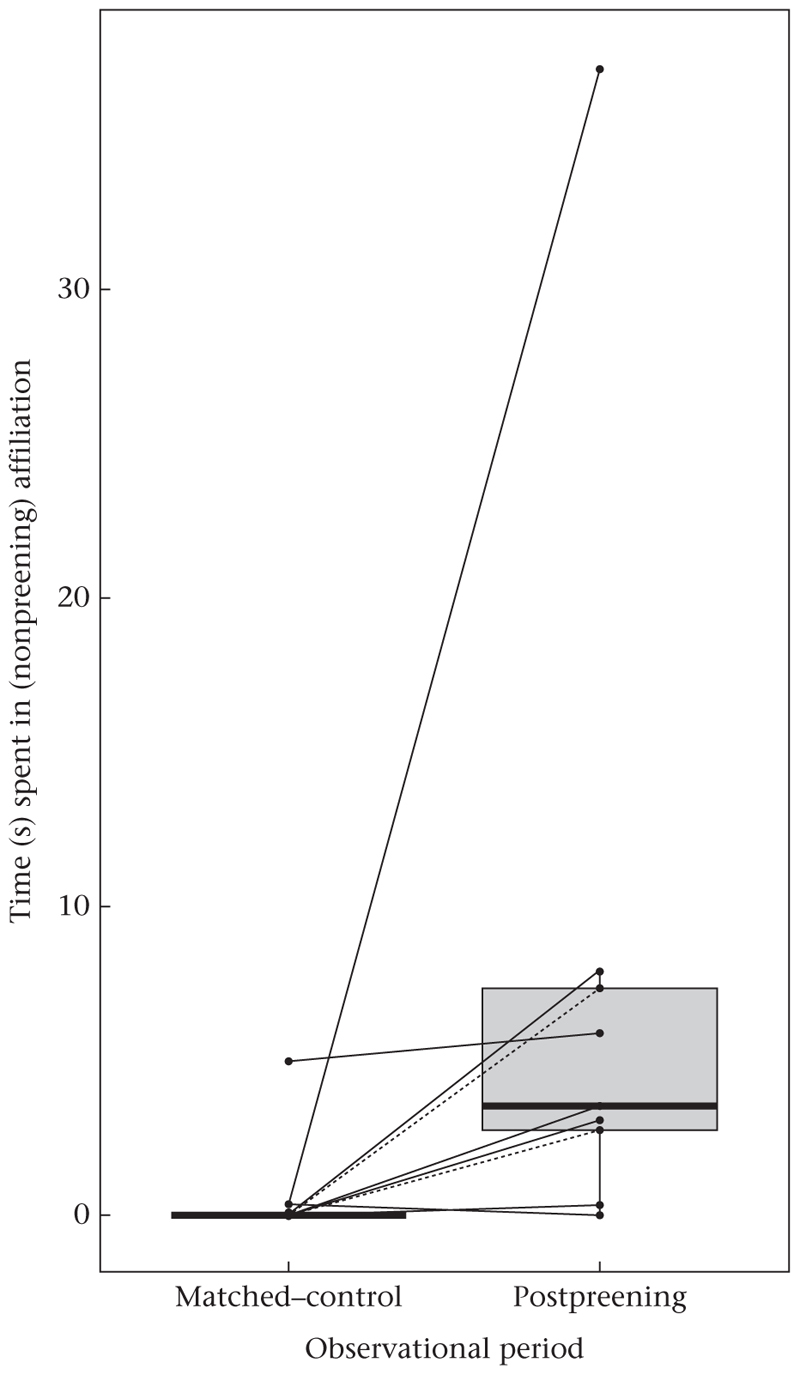
Time spent in (nonpreening) affiliative interactions in postpreening and the respective matched-control observations. Points represent the average time (s) per focal subject in each observation. The box plots show the median and 25th and 75th percentiles; the whiskers indicate the values within 1.5 times the interquartile range. Solid lines indicate female focal subjects; dashed lines indicate male focal subjects. Wilcoxon test: *P* = 0.012, *N* = 9.

**Figure 4:**
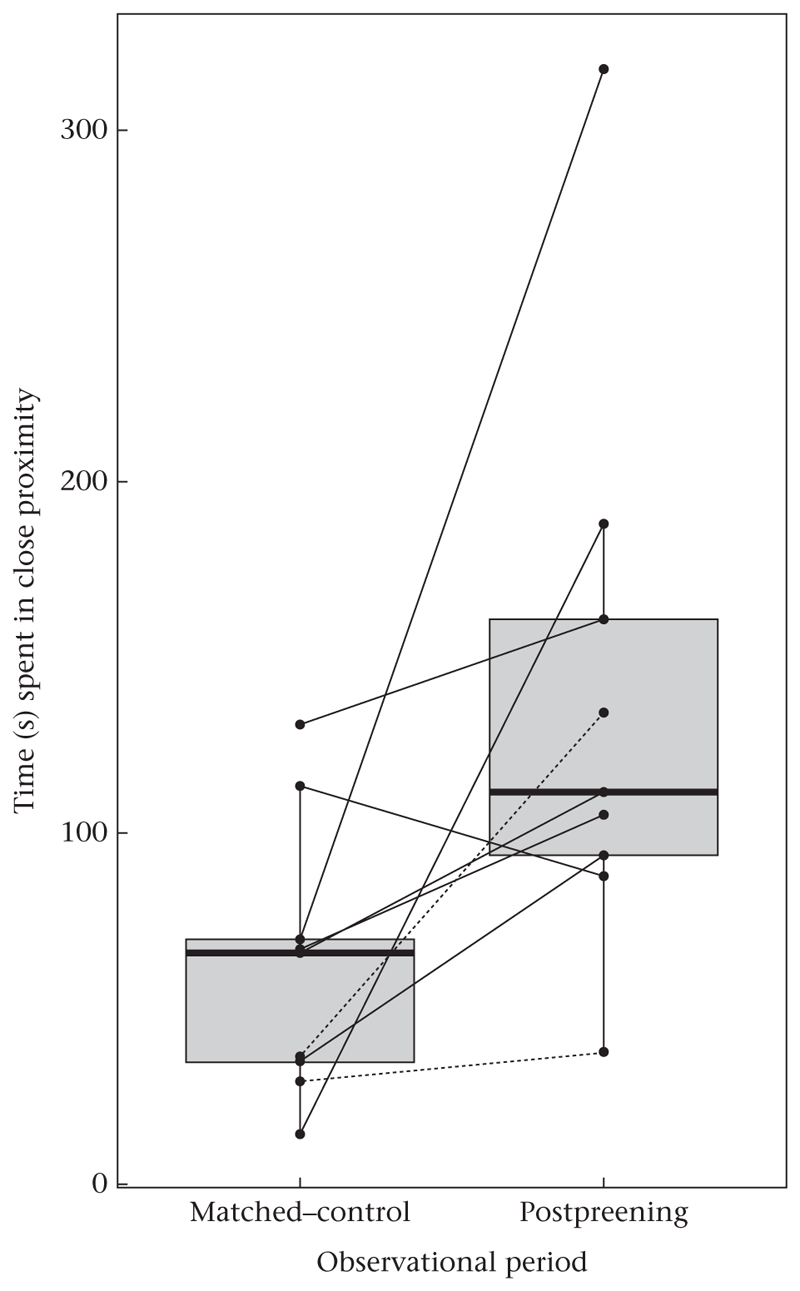
Time spent close to (< 1 m) at least one conspecific in postpreening and the respective matched-control observations. Points represent the average time (s) per focal subject in each observation. Solid lines indicate female focal subjects; dashed lines indicate male focal subjects. Wilcoxon test: *P* = 0.012, *N* = 9.

Focal subjects engaged in (nonpreening) affiliative behaviours, i.e. contact-sit and hold/touch other’s beak, for significantly longer in PP than MC observations (two-tailed Wilcoxon signed-rank test on paired samples: *V* = 43, *P* < 0.05, effect size *r* = 0.81, *N* = 9; median: PP: 3.52 s; MC: 0 s; Fig. 3) and spent significantly longer close to at least one other conspecific during the PP than the MC (two-tailed Wilcoxon signed-rank test on paired samples: *V* = 43, *P* < 0.05, effect size *r* = 0.81, *N* = 9; median: PP: 111.73 s; MC: 65.75 s; Fig. 4). No significant differences between PP and MC observations occurred when evaluating the number of approaches towards conspecifics (two-tailed Wilcoxon signed-rank test on paired samples: *V* = 31, *P* = 0.08, *N* = 9; Appendix Fig. A1) or the number of agonistic interactions (two-tailed Wilcoxon signed-rank test on paired samples: *V* = 5, *P* = 0.59, *N* = 9; Appendix Fig. A2).

### Self-directed Behaviours

No significant differences were found between PP and the associated MC observations for autopreening (two-tailed Wilcoxon signed-rank test on paired samples: *V* = 26, *P* = 0.294, *N* = 9; Appendix Fig. A3) or for nonpreening self-directed behaviours (the remaining self-directed behaviours: beak wipe, scratch, stretch, shake; two-tailed Wilcoxon signed-rank test on paired samples: *V* = 38, *P* = 0.076, *N* = 9; Appendix Fig. A4).

## Discussion

In the present study, we tested whether common ravens showed signs of contagion after witnessing conspecifics engaging in positive social interactions, namely allopreening. Focal subjects were more likely to engage in allopreening themselves in the PP observation phase than in the MC observation phase; in addition, they also showed more (nonpreening) affiliative interactions and spent more time close to at least one conspecific. Witnessing others in positive social interactions therefore led not only to behavioural matching, as expected by behavioural contagion, but also to a general increase in positive social interactions, as expected by emotional contagion. However, engaging in allopreening-related activities could also be interpreted as incomplete expressions of allopreening attempts. Hence, this observational study demonstrates a clear contagion effect in allopreening but remains vague in respect to the underlying mechanism.

That ravens can show emotional contagion has recently been experimentally demonstrated using a judgement bias paradigm (Adriaense et al., 2019). In this study, ravens were tested in a cognitive bias task before and after they had seen a conspecific expressing behaviours indicative of a positive or negative emotional state. Bystanders hardly expressed any indicative behaviours but showed a pessimistic judgement in the cognitive bias task after they had seen the conspecific in a negative emotional state. However, the study did not find evidence for a similar transfer of positive emotional states, possibly because of methodological constraints in the test set-up or because positive contagion is restricted to young birds and/or specific contexts. Indeed, positive emotional contagion has so far only been shown in young ravens in the context of play (Osvath & Sima, 2014; Wenig et al., 2021). In these studies, bystander birds engaged in locomotion and social play after seeing a conspecific manipulating objects (i.e. object play), indicating that they got into a general mood to play. The present study adds another context in which positive contagion could be studied in birds of several age classes. Following the argumentation introduced by Osvath and Sima (2014) for play, the protocol allows us to differentiate between behavioural and emotional contagion based on the selective matching of behaviours, as an indicator of behavioural contagion versus the expression of behaviours of different motor patterns, indicative of a given emotional state.

Recently, Berthier and Semple (2018) adapted the postconflict/matched-control method from de Waal and Yoshihara (1983) to study grooming contagion in Barbary macaques (more recently: Ostner et al., 2021). To our knowledge, our study is the first to apply this innovative approach to birds. We collected observational data on bystander behaviour only for a maximum of 10 min (Berthier & Semple, 2018:1 h, Ostner et al., 2021: 20 min). This relatively short observation time was informed by our previous studies on raven contagion and the general assumption that (emotional) contagion is a spontaneous, automatic reaction of the brain (action perception model: Preston & De Waal, 2002; Panksepp, 2005), making a quick response likely. The restriction of the observation time may have also led to the high percentage of ‘neutral’ PP/MC pairs (where allopreening did not occur in either the PP or MC observation phase) in the present study compared to previous studies on macaques (Berthier & Semple, 2018: 9.7%; Ostner et al., 2021: 8.5%; present study: 47.5%). Alternatively, the overall contagion effect of allopreening in ravens could be rather different from the contagion effect in primates. However, when comparing the non-neutral PP/MC pairs, six of nine focal subjects showed a higher percentage of ‘attracted’ than ‘dispersed’ PP/MC pairs, showing the same key pattern as in previous studies on macaques (Berthier & Semple, 2018; Ostner et al., 2021).

Ravens in our study did not show differences in (preening or nonpreening) self-directed behaviours or agonistic interactions when we compared PP and MC observations. The result on auto-preening is particularly interesting, as we originally considered this to be a marker for behavioural contagion. As witnessing others’ allopreening affected the ravens’ likelihood of engaging in allopreening (and other social behaviours) themselves but not autopreening, ravens may copy the other’s behaviour according to context (allopreening as a social interaction versus autopreening as self-maintenance). Notably, the contagion effect seemed to be based on the interaction type (sociopositive interaction), rather than a particular behaviour (preening). Alternatively, the effect could be due to differences in the emotional valence underlying allo- and autopreening. Increased levels of self-directed behaviours like autopreening have been suggested as indicative of coping with (mild) stress in several species, including ravens (Castles & Whiten, 2010; Massen, Pašukonis, et al., 2014; Sachs, 1988; Smolinsky et al., 2009). Although the current data do not provide any indication that bystander ravens were stressed, future studies should consider possible differences in valence when testing for the contagious nature of autopreening. The lack of significant differences in agonistic interactions between PP and MC could be due to our limited number of observations and restricted observation time, as we recorded few instances of agonistic interactions in the given time frame of 10 min. However, the finding that Barbary macaques also did not differ with regard to agonistic interactions in a comparison of postgrooming versus matched-control observations (Berthier & Semple, 2018) could strengthen the argumentation that the contagion effect in the context of allopreening is valence specific.

Another interesting path to consider in future studies is the role of social factors in positive contagion, such as hierarchies and relationships. High-ranking rhesus macaques have been shown to express allogrooming contagion more rapidly than low-ranking individuals, possibly due to lower constraints associated with their social status (Ostner et al., 2021). Comparably, nonbreeder ravens also rely on a linear dominance hierarchy from as young as 5 months postfledging (Loretto et al., 2012). They also form strong social bonds, which influence group structure and dynamics (Braun & Bugnyar, 2012), they keep track of their own and others’ social bonds (Boeckle & Bugnyar, 2012; Massen, Pašukonis, et al., 2014) and selectively intervene in bonding attempts of conspecifics (Massen, Szipl, et al., 2014). In the context of preening contagion, one could argue that ravens might be more suggestible to the behaviours and emotional states of closely bonded individuals (compare Jeon et al., 2010; Jeon & Shin, 2011; Langford et al., 2006; Martin et al., 2015) and would be more likely to pick up and align with their emotional states. However, if closely bonded allies were seen allopreening with a third party, observers could also perceive the affiliative interaction as rather stressful and threatening for their own bond and therefore intervene agonistically. As ravens have been reported to intervene in others’ bonding attempts (Massen, Szipl, et al., 2014), it might be difficult to distinguish whether allopreening interactions during PP phases were a consequence of contagion or an attempt to re-establish the social relationship when focal subjects engaged in affiliative contacts with one of the previous demonstrators. However, in the present data set, only one third of allopreening interactions in attracted pairs appeared between focal subjects and previous demonstrators while in the majority of cases focal subjects engaged in allopreening with an individual that had not been involved in affiliative interactions before.

In addition, or in interaction with social factors, future research should also assess the role of sex, age and personality traits (e.g. individual sociability) in animal studies on emotional contagion (e.g. compare Christov-Moore et al., 2014; Eisenberg & Lennon, 1983; Hein & Singer, 2008; Koski, 2014; Mikosz et al., 2016). However, assessing individual and social factors in the context of allopreening contagion was beyond the scope of this study as it requires a larger sample size and detailed information on the social structure within the group. Observations on our marked population of free-ranging ravens could thus be a next step.

Taken together, applying the PP/MC methodology offers a promising noninvasive way to study contagion in social animals within a positive context. It also provides a way to disentangle behavioural and emotional aspects of contagion. If subjects within PP observations showed increased rates only of allopreening, one could argue for the expression of behavioural matching without the subject’s emotional state necessarily being impacted; however, if subjects showed additional affiliative interactions (e.g. contact-sit, spending time in close proximity) and reduced rates of agonistic and/or (nonpreening) self-directed behaviours in PP observations, witnessing others in positive social interactions seems to have a more general influence on the bystander’s emotional state. In the present study, we found behavioural allopreening contagion as a response to observing conspecifics in allopreening interactions, providing observational evidence for behavioural contagion within a positive context in subadult ravens. We also found increased rates of other affiliation indicators, but no indication of complementary nonaffiliative behaviours, leaving the interpretation in respect to emotional contagion open.

## Supplementary Material

Appendix
