# Supplementary material for: Testing the contagious nature of allopreening: bystander ravens are affected by conspecifics’ affiliative interactions
Source: Anim Behav. Author manuscript; Available in PMC 2024 Jul 26. (PMC7616304; doi:10.1016/j.anbehav.2021.12.009)
Supplement: Appendix [file EMS197395-supplement-Appendix.pdf]

Appendix

Table A1
Ethogram

Behavioural category	Behaviour	Measurement unit	Description
Self-directed	Beak wipe	Frequency, one beak wipe on one side counts as one instance	Bird wipes its beak against a hard surface, typically a perch
Self-directed	Scratch	Frequency, two bouts are separated by 5 s	Bird scratches any body part with its foot
Self-directed	Stretch	Frequency, two bouts are separated by 5 s	Bird stretches its wings and legs in the air
Self-directed	Shake	Frequency, two bouts are separated by 5 s	Bird shakes its body and feathers
Self-directed	Autopreening	Duration	Bird holds and/or gently runs its feathers through its beak
Agonistic	Chase	Duration*	Bird pursues another one in flight/on ground
Agonistic	Displace	Frequency	Bird retreats/moves away within 1–2 s after another bird approaches to within 1 m and eventually takes its position. With or without vocalization of either bird, and with or without physical contact between them
Agonistic	Threat	Frequency	Bird threatens another one, with display 'thick head approach' and / or visual threat (the aggressor pecks in the direction of the victim without touching it)
Agonistic	Fight	Duration*	Two birds hit each other with their feet/beaks, with one/both birds jumping in the air or with one bird on the ground and one sitting on top
Affiliative	Contact-sit	Duration	Two birds sit next to each other within 10 cm, typically on a perch
Affiliative	Touch/hold	Duration	Bird touches another's body with its beak, without performing preening movements
Affiliative	Allopreening	Duration	Bird touches/runs its beak through the feathers of another bird for longer than 2 s
Affiliative	Approach	Frequency, two bouts are separated by 5 s	Bird actively moves close to a conspecific, followed by a positive (affiliative) or neutral (no) interaction
Affiliative	Close proximity	Duration	Bird spending time within 1 m of conspecifics

* As 'chase' and 'fight' occurred only once (and for less than 5 s) during data collection, we treated these behaviours as frequency data and pooled them with 'displace' and 'threat' in the category 'agonistic behaviours'.

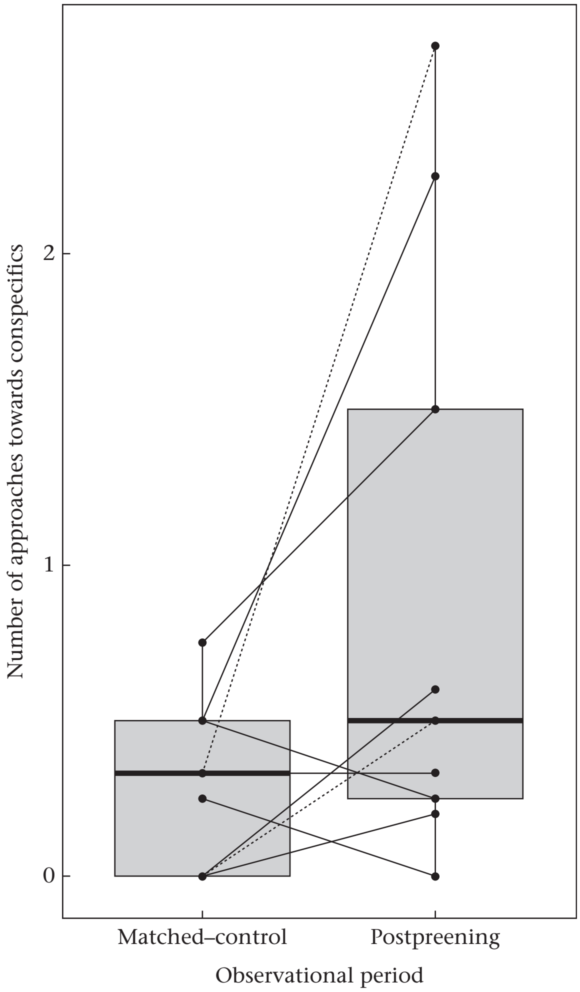

Figure A1. Number of approaches towards at least one conspecific in postpreening and the crespective matched-control observations. Points represent the average number per focal subject in each observation. The box plots show the median and 25th and 75th percentiles; the whiskers indicate the values within 1.5 times the interquartile range. Solid lines indicate female focal subjects; dashed lines indicate male focal subjects. Wilcoxon test: $P = 0.08$, $N = 9$.

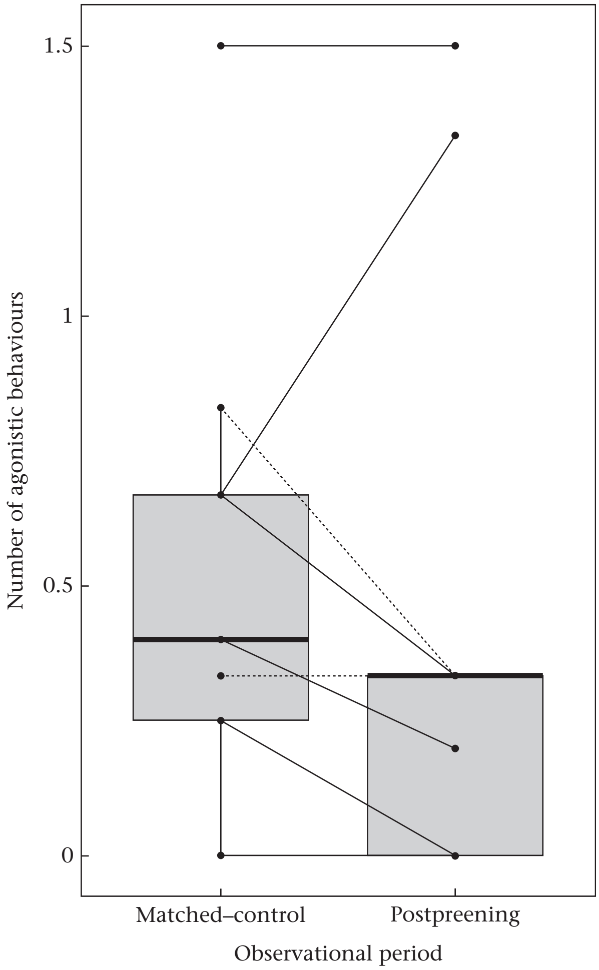

Figure A2. Number of agonistic behaviours in postpreening and the respective matched-control observations. Points represent the average number per focal subject in each observation. The box plots show the median and 25th and 75th percentiles; the whiskers indicate the values within 1.5 times the interquartile range. Solid lines indicate female focal subjects; dashed lines indicate male focal subjects. Wilcoxon test: $P = 0.59$, $N = 9$.

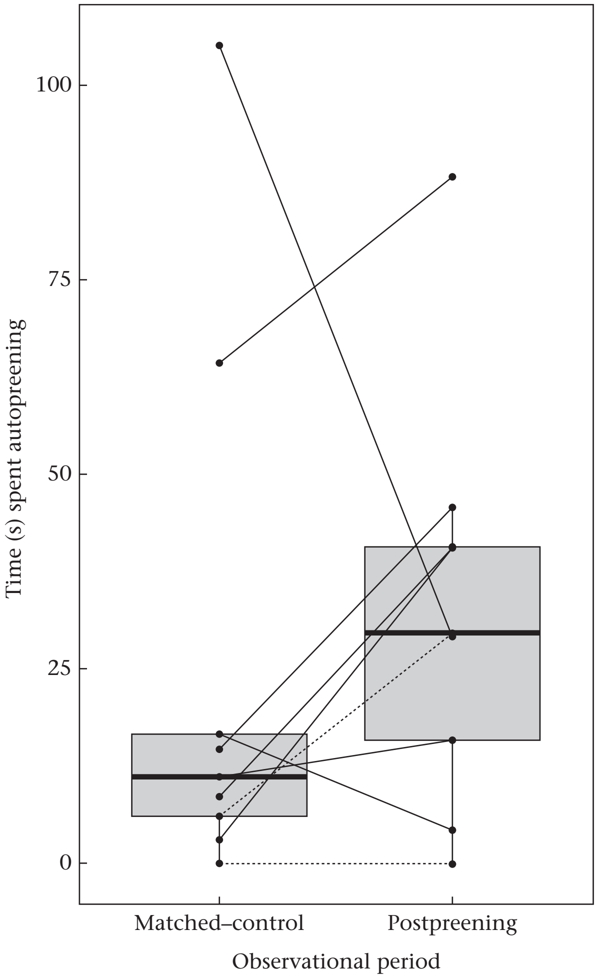

Figure A3. Time spent autopreening in postpreening and the respective matched-control observations. Points represent the average time (s) per focal subject in each observation. The box plots show the median and 25th and 75th percentiles; the whiskers indicate the values within 1.5 times the interquartile range. Solid lines indicate female focal subjects; dashed lines indicate male focal subjects. Wilcoxon test: $P = 0.29$, $N = 9$.

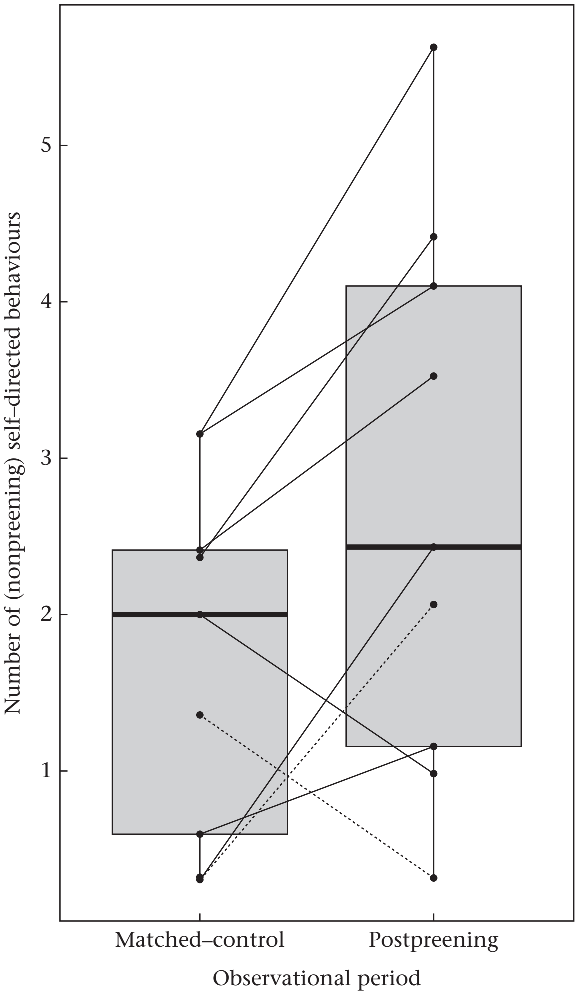

Figure A4. Number of (nonpreening) self-directed behaviours in postpreening and the respective matched-control observations. Points represent the average number per focal subject in each observation. The box plots show the median and 25th and 75th percentiles; the whiskers indicate the values within 1.5 times the interquartile range. Solid lines indicate female focal subjects; dashed lines indicate male focal subjects. Wilcoxon test: $P = 0.074$, $N = 9$.
